# Analysis of the Temporal Patterning of Notch Downstream Targets during *Drosophila melanogaster* Egg Chamber Development

**DOI:** 10.1038/s41598-020-64247-2

**Published:** 2020-04-30

**Authors:** Molly Rowe, Lily Paculis, Fernando Tapia, Qiuping Xu, Qian Xie, Manyun Liu, Allison Jevitt, Dongyu Jia

**Affiliations:** 1 Department of Biology, Georgia Southern University, Statesboro, GA 30460 USA; 2 Morphism Institute, Seattle, WA 98117 USA; 3 Department of Biostatistics, Epidemiology and Environmental Health Sciences, Jiann-Ping Hsu College of Public Health, Georgia Southern University, Statesboro, GA 30460 USA; 4 Department of Biological Science, Florida State University, Tallahassee, FL 32306-4370 USA

**Keywords:** Gene expression analysis, Pattern formation

## Abstract

Living organisms require complex signaling interactions and proper regulation of these interactions to influence biological processes. Of these complex networks, one of the most distinguished is the Notch pathway. Dysregulation of this pathway often results in defects during organismal development and can be a causative mechanism for initiation and progression of cancer. Despite previous research entailing the importance of this signaling pathway and the organismal processes that it is involved in, less is known concerning the major Notch downstream targets, especially the onset and sequence in which they are modulated during normal development. As timing of regulation may be linked to many biological processes, we investigated and established a model of temporal patterning of major Notch downstream targets including *broad*, *cut*, and *hindsight* during *Drosophila melanogaster* egg chamber development. We confirmed the sequential order of Broad upregulation, Hindsight upregulation, and Cut downregulation. In addition, we showed that Notch signaling could be activated at stage 4, one stage earlier than the stage 5, a previously long-held belief. However, our further mitotic marker analysis re-stated that mitotic cycle continues until stage 5. Through our study, we once again validated the effectiveness and reliability of our MATLAB toolbox designed to systematically identify egg chamber stages based on area size, ratio, and additional morphological characteristics.

## Introduction

Biological organisms require complex signaling interactions and coordination of these interactions to survive and reproduce. Through cellular signaling pathways, cells interact and exchange information with neighboring cells, ultimately determining cell fate and organismal development as a whole^1^. Of these often complex signaling networks, the Notch signaling pathway is one of the most evolutionarily conserved^2^. This pathway facilitates local cell-cell interactions to control cell fate and plays a role in many aspects of development, including cell self-differentiation and self-renewal^3,4^.

As demonstrated by much previous research, the Notch pathway is necessary for proper cellular and organismal development. However, defects or absence of proper functions in this pathway often lead to problems with development, and often certain types of cancers^5^. Interestingly, the Notch pathway has been linked to not only tissue growth and tumorigenesis, but also cell death and tumor suppression in some circumstances^6^. Although this highlights the complexity of the function of the Notch pathway, recent research has indicated that the core molecular design is actually rather simple. In canonical Notch signaling, a transmembrane ligand Delta on an adjacent cell interacts with a receptor Notch on the cell receiving the signal, which then results in the receptor being cleaved to release the Notch intracellular domain (NICD). NICD moves to the nucleus of the cell, where it later interacts with DNA-binding proteins to begin transcription of the appropriate genes^7^.

As with many other aspects of development, recent research has shown that the timing in which this Notch signaling induces expression of its target genes is crucial for many aspects of organismal development. For instance, a study identified that interactions between epithelial cells in mice require proper sequential interaction of signaling pathways, among these most notably, the Notch pathway. This research also showed the transcription and expression patterns of various Notch components, suggesting that timing may play a role in the interactions of downstream targets^8^. In *Drosophila*, researchers also reported the importance of temporal regulation of neuroblasts for the cell fate and survival of Notch-mediated neurons^9,10^. During *Drosophila* oogenesis, the follicle cells of egg chambers sequentially undergo three different cell cycle programs: the mitotic cycle (stages 1-5), endocycle (stages 6-10a), and gene amplification (stages 10b-14), which are considered as early oogenesis, midoogenesis and late oogenesis, respectively^11^. The Notch pathway is the main signaling pathway to regulate the switches of cell cycles. There are many important downstream targets of Notch signaling, including *cut*, *hindsight (hnt)* and *broad (br)*, during egg chamber development^12–14^. Cut is expressed in the mitotic cycle, then inhibited by Notch signaling in the endocycle. Opposite to the expression pattern of Cut, Hnt and Br are absent in the mitotic cycle, and upregulated by Notch signaling in the endocycle. In addition, Hnt and Br can promote each other, but repress Cut^12–14^. Scientists frequently use Cut expression for the mitotic cycle identification, and Hnt for the endocycle identification. However, an intermediate stage between the mitotic cycle and endocycle has been identified; when Hnt and Cut overlap expression^15^. We also observed similar overlapping expression patterns, and suggested that anterior follicle cells might undergo the mitotic cycle/endocycle (M/E) switch earlier^16^. In addition, as a direct downstream target of Notch signaling in follicle cells, the protein level of Br is highly upregulated at stage 6, but we reported that weak Br could be detected at stage 5 egg chambers based on morphology^14^. In summary, previous studies have shown that certain downstream targets of the Notch signaling pathway respond differently and at different times to stimuli. The findings highlight the sensitivity of the downstream targets *br, cut*, and *hnt* to Notch signaling during egg chamber development, indicating that the timing in which gene expression appeared in egg chambers sometimes varied.

Here, we present a model of the temporal patterning of Notch downstream targets *br, hnt* and *cut* during *Drosophila melanogaster* egg chamber development. Investigation and establishment of this signaling model during normal development may serve as a baseline for comparison of additional research findings, as modulation of such downstream targets is often accompanied by cell-cycle switches. In addition, we further confirmed the validity of a MATLAB toolbox for identification of egg chamber stages based on area size, ratio, and additional morphological characteristics that improves accuracy of stage identification, reduces reliance on visual determination, and can be useful for many areas of research.

## Results

### Gene expression pattern during transitional stages

**Figure 1:**
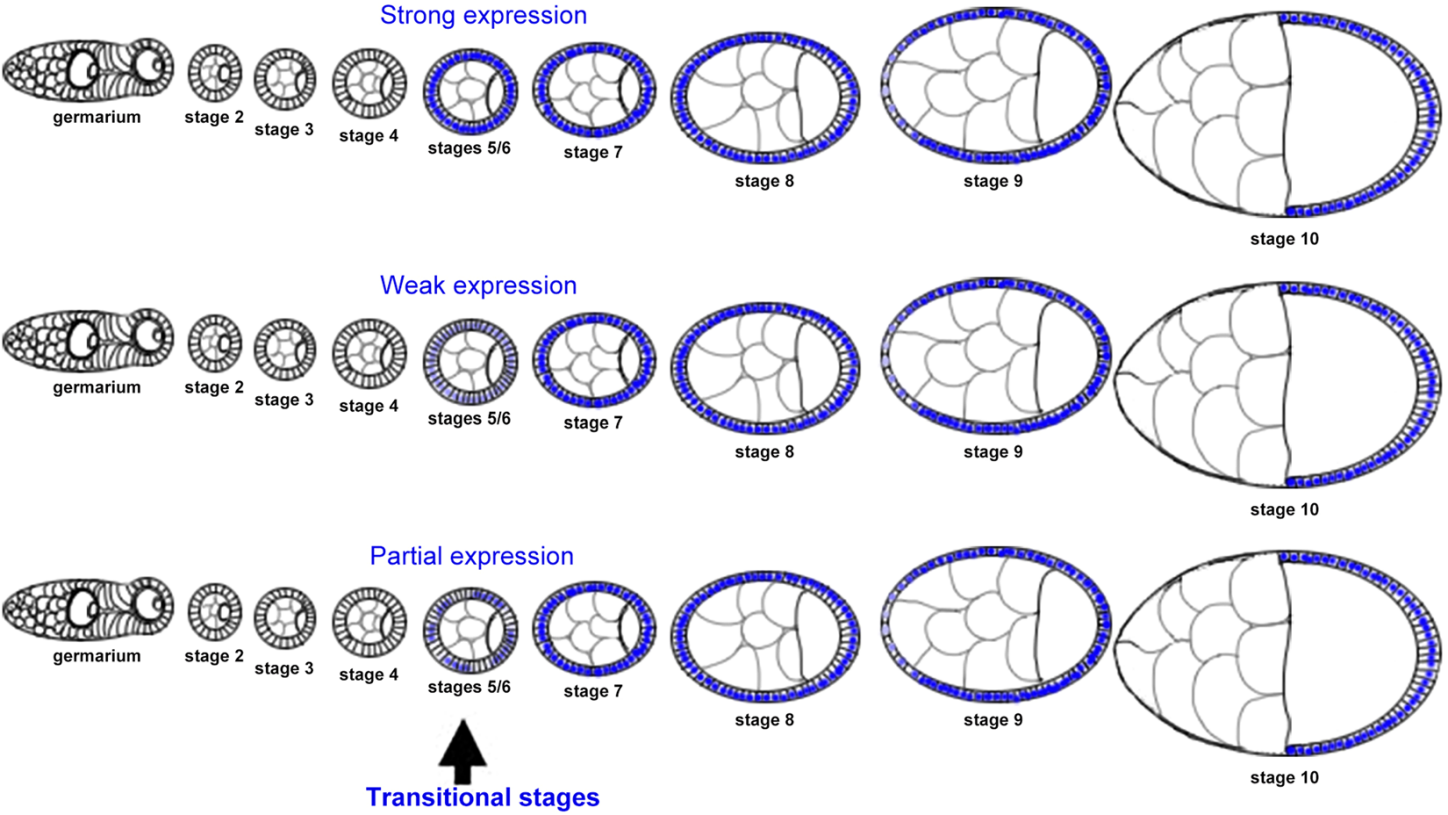
Development of *Drosophila* egg chambers from early to late stages with theoretical protein expression levels indicated in blue. Strong, weak, and partial expression of Br are shown as an example.

Our previous findings with *Drosophila* follicle cells showed the expression of Hnt and Cut overlapped and weak Br could be detected at stage 5 egg chambers, suggesting varied sensitivity of *br, cut*, and *hnt* to Notch signaling^11,14,16^. As indicated in Fig. 1, we frequently observed strong gene expression based on immunohistochemical staining using antibodies. There is an inherent emphasis on the strong levels of gene expression when interrogating gene function or developmental patterning. In fact, from no expression to strong expression, or vice versa, there are transitional stages of gene expressions, during which the genes are weakly or partially expressed. Transitional stages are frequently unnoticed, but can be informative to indicate signaling onset. Focusing on more nuanced transitional patterns of expression might also provide additional mechanistic insight into genetic regulation.

It is known that *br* and *hnt* are activated by Notch signaling during the mitotic cycle, while *cut* is suppressed by Notch in the later endocycle. This marks the mitotic to endocycle transition (also called the M/E switch). However, the exact stages of their upregulation/downregulation are still debatable, partially because of different staging methods and inconsistent individual criteria. Previously, we created a toolbox to unbiasedly identify the stages based on morphological characteristics marked by DAPI staining. We concluded that follicle cells underwent the mitotic cycle at stage 5, and endocycle is from stage 6 without mitosis^11,17^.

**Figure 2:**
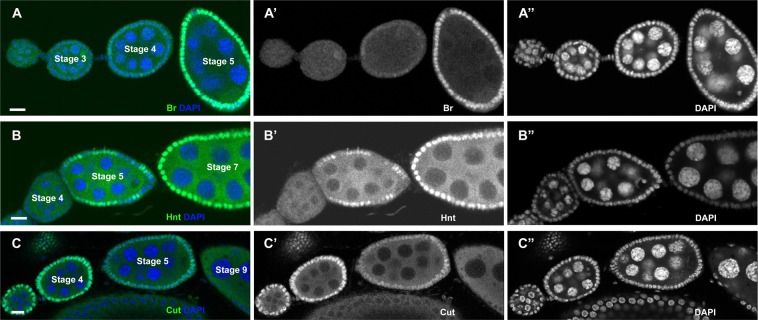
Confocal images of *Drosophila* egg chambers with stages identified. No expression, weak/partial expression, and full expression are shown for each target of interest. Downstream targets Br and Hnt are shown in earlier and later stage egg chambers corresponding with no expression, transitional, and later stage full expression. Cut is shown with early stage full expression, transitional, and later stage no expression. Scale bar =10 µm.

We were particularly interested in the transitional expression patterns, because they indicated the sensitive response of gene activation/inactivation and signaling onset. To characterize these patterns, we specifically selected egg chambers during the transitional stages, which showed weak/partial upregulation of Br and Hnt, and downregulation of Cut (Fig. 2).

### Early gene upregulation/downregulation during transitional stages

**Figure 3:**
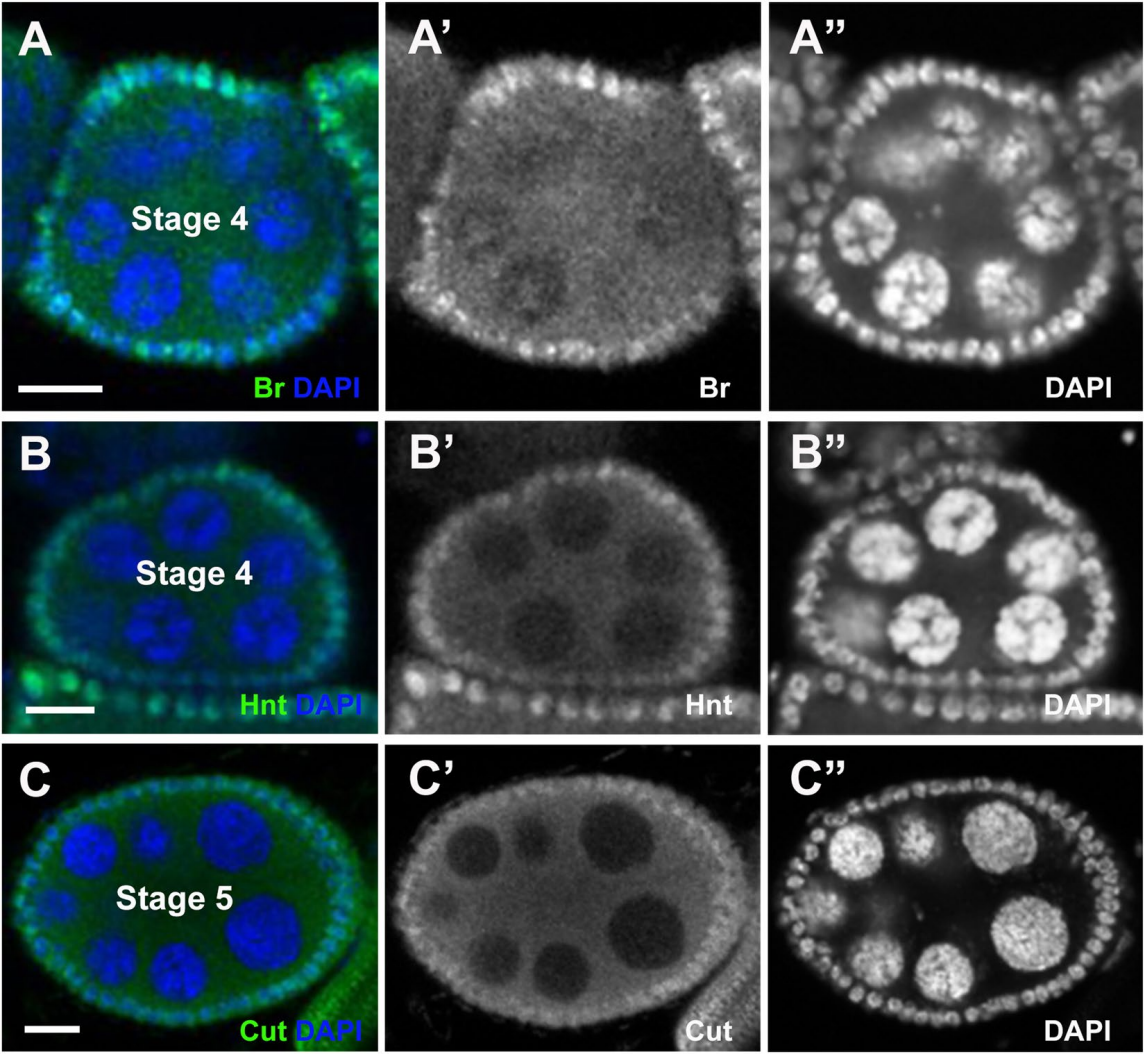
Br and Hnt appear as early as stage 4, while Cut decreases at stage 5. Confocal images showed antibody staining in green and DAPI in blue. Images were obtained via confocal microscopy and stage assignment was conducted using the MATLAB toolbox and confirmed visually. Scale bar =10 µm.

Previously, it was known that Notch signaling starts from stage 5^18,19^, which then activates Br at stage 5/6^14^, and Hnt at stages 6/7^13,20^. Downregulation of Cut happens at stages 6/7^12,21^. However, surprisingly, we found that early activation of both Br and Hnt could be observed as early as stage 4 (Figs. 3A,B), which was clearly indicated by the 5-blob phenotype of nurse cells in the egg chambers. During the *Drosophila* egg chamber development, the chromosomes of nurse cells appear polytene in stages 2–4 but later on dissociate^22^. For a stage 5 egg chamber, nurse cells no longer contain polytene chromosomes^11,17^. Prior to the breakdown of polytene chromosomes, the nurse cell chromosomes present a striking 5-blob phenotype, which can be used as a hallmark for stage 4 egg chambers^11,17^. In addition, Cut was not downregulated at stage 4, but its early downregulation could be found at stage 5 (Fig. 3C).

### Sequential gene expression pattern during transitional stages

**Figure 4:**
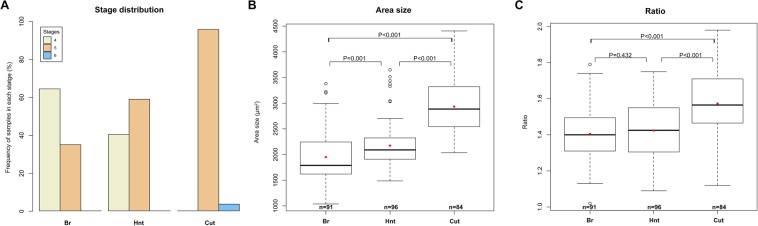
Statistical analysis of data obtained from egg chamber staging of transitional Br, Hnt, and Cut expression. Stage distribution (**A**), area size (**B**), and ratio (**C**) of egg chambers for each target are shown.

Immunostaining is mainly used to examine the expression patterns of target proteins. Primary antibodies are raised in various species, and those raised in the same host species can’t be stained simultaneously because the secondary antibodies can’t distinguish them. Host species of primary antibodies mouse anti-Cut (2B10)^23^, mouse anti-Br-Core (25E9)^24^, and mouse anti-Hnt (1G9)^25^ from Development Studies Hybridoma Bank were the same, therefore co-staining of them cannot be achieved to show the sequential gene expression patterns in the same egg chambers. Our previously developed toolbox can automatically identify egg chamber stages based on egg chamber size and ratio^11^. We systematically analyzed all the results of the transitional expression patterns based on confocal images using the toolbox. We found that transitional Br was mostly expressed in stage 4 egg chambers (64.8%, n = 91), while transitional Hnt was only found in 40.6% (n = 96) stage 4 egg chambers (Fig. 4A). Our findings suggested that Br was upregulated earlier than Hnt. In addition, the transitional expression patterns of Cut primarily occurred in stage 5 (96.4%, n = 84), indicating Cut was downregulated after the upregulation of Br and Hnt (Fig. 4A).

The DAPI images were collected from the largest cross-sectional area of the egg chambers in the middle plane, and this area size was used for our stage identification^11^. We found that egg chambers with transitional Hnt-expressing were significantly larger than those of transitional Br-expressing egg chambers, while the area sizes of both Hnt- and Br-expressing egg chambers were significantly smaller than those of transitional Cut-expressing egg chambers (Fig. 4B), further confirming the Br-Hnt-Cut sequential order of expression. We also examined the ratio of the major and minor axes of egg chambers, and found that transitional Cut-expressing egg chambers were more significantly elongated than Hnt- and Br-expressing egg chambers (Fig. 4C), indicating transitional Cut-expressing egg chambers were at later stages. Hnt- and Br-expressing egg chambers were similar in ratio (Fig. 4C), which is consistent with our previous findings that ratio differences were not evident until stage 5^11^.

### Cell cycle pattern during transitional stages

**Figure 5:**
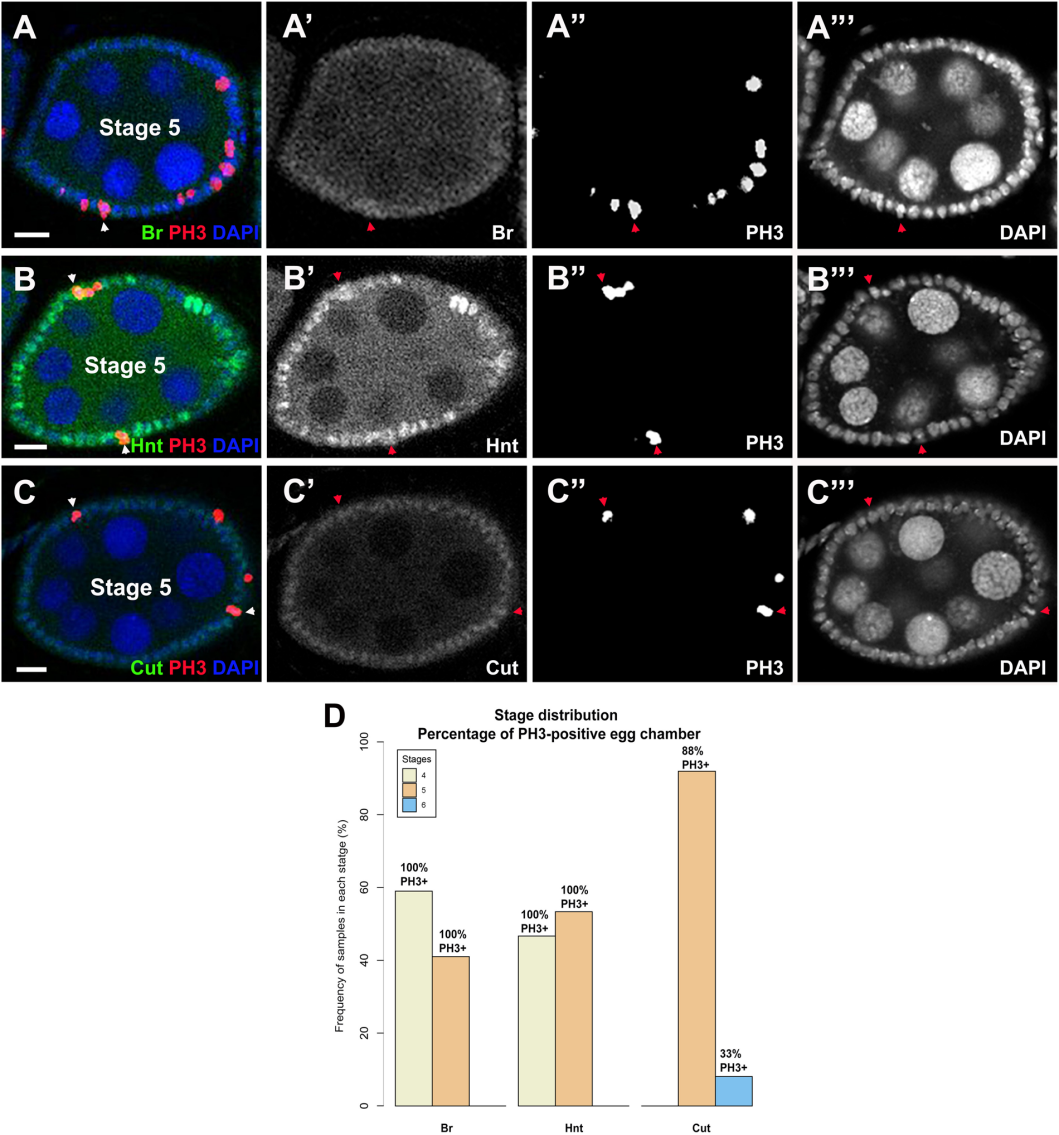
Co-staining of PH3 with Br, Hnt and Cut. (**A–C**) Confocal images showed the co-stainings, Br, Hnt and Cut antibody staining in green, PH3 in red and DAPI in blue. Co-expressing follicle cells were indicated by arrowheads. (**D**) Statistical analysis of data obtained from egg chamber staging of transitional Br, Hnt, and Cut expression with PH3 co-staining. Percentage of PH3-positive egg chambers were labeled on top of each column. Scale bar =10 µm.

We identified and focused on the specific transitional stages with weak or partial expression of Notch target genes. We would ask whether these transitional egg chambers have already entered endocycle, ceasing mitotic cycle. Phospho-histone H3 (PH3) has frequently been utilized as a mitotic marker^14,18^, which shows oscillating patterns in follicle cells during the mitotic cycle, but disappears once they enter the endocycle. We co-stained PH3 with Br, Hnt, and Cut, respectively. We systematically analyzed the co-staining results at the transitional stages based on confocal images using the toolbox. Consistent with prior findings (Fig. 4), We found that transitional Br was mostly expressed in stage 4 egg chambers (59%, n = 39), while transitional Hnt was only found in 46.7% (n = 30) stage 4 egg chambers (Fig. 5). The transitional downregulated expression of Cut primarily occurred in stage 5 (91.9%, n = 37), further confirming Cut was downregulated after the upregulation of Br and Hnt (Fig. 5). In addition, we found 100% of both Hnt- and Br-expressing egg chambers in stages 4 and 5 still showed mitotic marker PH3 (Fig. 5), suggesting early weak upregulation of Br or Hnt is not robust enough to cease the mitosis to enter endocycle. Further observation showed that some Hnt- and Br-expressing follicle cells were still in mitotic cycle (Figs. 5A,B), further confirming that mitotic cycle continues until stage 5.

## Discussion

Overall, the importance of the Notch pathway for many aspects of development has clearly been demonstrated by numerous publications, and the pathway itself has been studied extensively. The Notch pathway is essential for many diseases, including Zika virus disease^26^, cardiovascular diseases^27^, Down syndrome^28^, and various cancer types^29–31^. Therefore, better understanding of the complex Notch pathway from all aspects are needed for potential therapeutic values. While there has been extensive research entailing what the Notch signaling pathway does and what processes it may be directly and indirectly involved in, the temporal expression of its major targets relative to one another has not been extensively studied.

During *Drosophila* oogenesis, Notch signaling plays a vital role in cell cycle switches. Previous research has discovered several important downstream targets in this pathway, but comparatively little is known about the timing in which they are activated in normal developmental processes. As downstream targets may respond to Notch signaling at different times, insight into this activation sequence may have many implications for the field. We previously studied some important downstream targets of Notch signaling, including *cut*, *hindsight (hnt)* and *broad (br)*, during egg chamber development. In addition, the three targets also showed varied sensitivity to Notch. Therefore, we decided to examine these three important Notch targets to understand the temporal patterning. Immunostaining is the most popular way to examine the expression patterns of certain proteins. However, primary antibodies raised in the same host species can’t be stained simultaneously. In our case, we found the publicly available primary antibodies from Developmental Studies Hybridoma Bank. Unfortunately, the host species of primary antibodies of mouse anti-Cut (2B10), mouse anti-Br-Core (25E9), mouse anti-Hnt (1G9) were the same. To overcome this barrier, we applied a previously developed MATLAB toolbox to automatically and unbiasedly identify egg chamber stages based on egg chamber morphology. Using the toolbox, we were able to decipher the temporal expression patterns of Br, Hnt and Cut. The effectiveness of the toolbox also enabled our scientific community to study egg chamber temporal expression patterns, even though scientists might not have primary antibodies from different host species.

**Figure 6:**
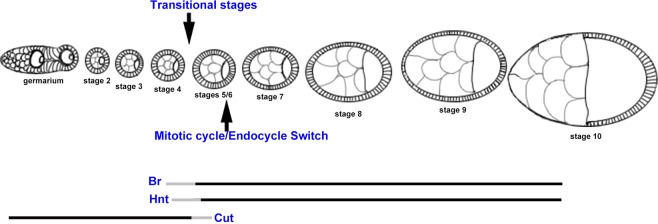
Model for *broad*, *cut*, and *hindsight* expression patterning during *Drosophila* egg chamber development. The model is based on statistical analysis of data acquired from egg chamber staging. Transitional stages for each target appear near stages 4, 5, and 6 of development with *broad* upregulation happening first, *hindsight* upregulation next, then *cut* downregulation occurring from stage 5. Our further mitotic marker analysis re-stated that mitotic cycle continues until stage 5.

Surprisingly, we found that both Br and Hnt were upregulated by Notch signaling as early as stage 4, and Br was upregulated earlier than Hnt (Fig. 4). Previously, it was well known that Notch signaling was activated by Delta ligand binding to Notch receptor at stage 5^18,19^. We speculate that there was a biased focus on strong expression of Notch signaling components to define the activation stages. Here, we focused on the specific transitional stages with weak or partial expression of target genes. With systematic analyses, we showed that Notch signaling could be activated at stage 4, one stage earlier than the stage 5, a previously long-held belief. In addition, downregulation of Cut takes place at stage 5, earlier than previously believed stages 6/7. Taken together, for the first time, we discovered that Notch signaling is activated at stage 4. As a direct transcriptionally regulated target of Notch, Br is upregulated at stages 4/5. Later than Br, Hnt is activated at stages 4/5. Due to the upregulation of Notch signaling, Cut expression is downregulated at stage 5. Our further mitotic marker analysis re-stated that mitotic cycle continues until stage 5 (Fig. 6). The observed variability in the weak/partial expression patterns at transitional stages might be caused by the unsynchronized Notch activation in the follicle cell. It might take time for inherent property of the cell cycle regulation system to finally transit the egg chamber into the endocycle at stage 6. It will also be interesting to study how the Notch signaling downstream targets interact with each other to boost the cell cycle transition at this special transitional stage. Further research into this aspect of Notch signaling may serve as a basis for additional investigation of developmental issues and defects. Better understanding of the complex Notch pathway and its downstream network could shed new lights on developmental regulations and cancer mechanisms.

## Materials and Methods

### Fly strains and genetics

Wild-type (*w*^*1118*^) *Drosophila melanogaster* flies were obtained from Bloomington *Drosophila* Stock Center (#5905), cultured with standard Bloomington medium and fed with yeast paste two days before dissection to enlarge ovaries. Female *Drosophila* were anesthetized and dissected according to standard techniques^32^.

### Immunohistochemistry and image acquirement

Immunohistochemistry was performed according to previous description^32^. The primary and secondary antibodies used in this study were as follows: mouse anti-Cut (2B10) (1:50; Developmental Studies Hybridoma Bank, USA), mouse anti-Br-Core (25E9) (1:30; Developmental Studies Hybridoma Bank, USA), mouse anti-Hnt (1G9) (1:15; Developmental Studies Hybridoma Bank, USA), rabbit anti-PH3 1:200 (Upstate Biotechnology, NY, USA) and Alexa Fluor secondary antibodies (1:400; Invitrogen). To stain nuclei, DAPI (1:500; Invitrogen) was applied. All samples were imaged using a Zeiss LSM 710 confocal microscope at Georgia Southern University. The acquired DAPI images represented the largest cross-sectional area of the egg chambers in the middle plane. Images were obtained of egg chambers showing partial, weak, strong and no expression of the targets of interest.

### Image processing methods

Images of DAPI staining were formatted using the Fiji package from Image J, and the images were cropped to display a single egg chamber. Egg chamber images were further analyzed using scripts written in MATLAB, as previously described^11^. This toolbox was used to automatically identify egg chamber stages based on egg chamber size and ratio. Egg chamber size was the size of the largest cross-sectional area of the egg chambers in the middle plane, and was measured in μm^2^. The egg chamber ratio was the ratio of the major and minor axes. Visual determination of egg chamber stage based on morphological criteria was used to confirm toolbox findings^11,17^.

### Quantitative methods

Statistical analyses were performed based on egg chamber staging data. Boxplots were used to display the distribution of data in Fig. 4. Mean values of each group were indicated by the red dots in the figure. The stage distribution plot in the Fig. 4 showed the frequency of specimens in each stage (from 4–6) in Br, Hnt and Cut groups. Beside the visualization, pairwise permutation tests were also conducted to assess the differences of the group-wise means. To control the familywise error rate, Bonferroni correction was further applied^33^. Since there were 3 tests in each table, instead of setting the critical P value for significance to 0.05, we use a lower critical value 0.017 (0.05/the number of tests = 0.017). In the boxplot, we would only consider individual tests with P < 0.017 to be statistically significant.

## Data Availability

The authors declare that the main data of this study are available within the article.
